# Oleic Acid-Esterified Octacosanol as a Functional Ingredient to Counter Obesity-Associated Lipid Dysregulation through PPAR-Targeted Regulation

**DOI:** 10.1021/acs.jafc.5c10351

**Published:** 2025-11-18

**Authors:** Yen-Chun Koh, Sudthida Kamchonemenukool, Pin-Yu Ho, Monthana Weerawatanakorn, Min-Hsiung Pan

**Affiliations:** † Institute of Food Science and Technology, 33561 National Taiwan University, Taipei 10617, Taiwan; ‡ Department of Agro-Industry, Faculty of Agriculture, Natural Resources and Environment, 59212 Naresuan University, 99 Moo 9, Tha Pho, Phitsanulok 65000, Thailand; § Department of Medical Research, China Medical University Hospital, China Medical University, 40447 Taichung City, Taiwan; ∥ Department of Health and Nutrition Biotechnology, Asia University, 41354 Taichung City, Taiwan

**Keywords:** policosanol, octacosanol, esterification, oleic acid, lauric acid

## Abstract

Octacosanol, a major constituent of policosanol, exhibits lipid-lowering activity, particularly when esterified with fatty acids. Although its cholesterol-lowering actions have been linked to the modulation of fatty acid and cholesterol biosynthesis, its functions within adipose tissue remain poorly defined. Here, we examined nonesterified octacosanol (NO), lauric acid-esterified octacosanol (LEO), and oleic acid-esterified octacosanol (OEO) in high-fat diet-fed mice for 11 weeks. Target prediction and molecular docking identified PPARα and PPARδ as putative targets of octacosanol, guiding downstream mechanistic analyses in adipose tissue. Both NO and OEO enhanced lipolysis; NO preferentially increased fatty acid β-oxidation, whereas OEO specifically promoted thermogenic remodeling, indicating distinct metabolic consequences driven by ester chemistry. Together, these findings demonstrate that structural modification, particularly oleic acid esterification, substantially augments the metabolic activity of octacosanol in lipid catabolism and thermogenesis, underscoring its potential relevance in obesity-associated metabolic regulation.

## Graphical abstract

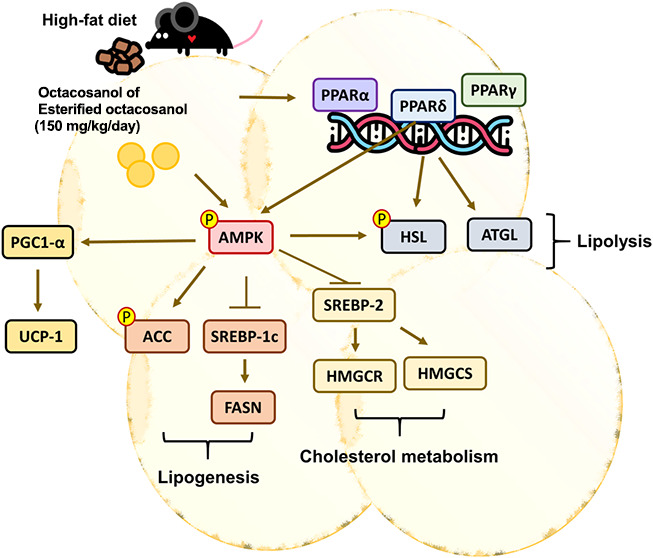

## Introduction

Policosanol (PC) is a natural mixture of aliphatic alcohols, typically extracted from sugar cane (*Saccharum officinarum* *L*.) wax, beeswax, rice bran, and wheat germ. It is also frequently obtained from byproducts of the food industry, particularly those generated during refining processes.^1^ The most prevalent PC in dietary plant is octacosanol (C-28, ∼60%) followed by triacontanol (C-30) and hexacosanol (C-26),^2^ and rice bran is considered one of the richest natural sources of octacosanol.^3^ The regulatory effects of octacosanol on lipid metabolism, cholesterol reduction, and energy enhancement have been previously reported.^4^ Consequently, it has been used in the development of functional foods and therapeutic applications. Importantly, as early as 1994, toxicity studies reported that the oral LD_50_ of octacosanol in rats was as high as 18 g/kg, indicating its safety and supporting its use as a functional component.^5^

PC is naturally present in esterified form; however, during extraction from natural sources, saponification using a hot alkaline solution results in the breakdown of esters into free fatty alcohols and fatty acids in the final extract.^6^ Previous studies have shown that oleic acid-esterified policosanols exhibit greater absorption than both nonesterified forms and butyric acid-esterified policosanols, indicating higher bioavailability.^7^ Moreover, oral administration of 164 mg/kg oleic acid-esterified octacosanol significantly reduced plasma total cholesterol and LDL-C levels in rats. In our previous study, we identified PC as naturally occurring components in rice bran oil and coconut oil.^8^ Given the abundance of oleic acid in rice bran oil and lauric acid in coconut oil, we esterified pure octacosanol with these fatty acids to evaluate their physiological benefits in comparison to nonesterified octacosanol.^8^ Surprisingly, oleic acid-esterified octacosanol (OEO) exhibited a greater inhibitory effect on hepatic lipid and cholesterol synthesis in HFD-fed mice compared to both lauric acid-esterified octacosanol (LEO) and nonesterified octacosanol (NO). Nonetheless, both NO and LEO also demonstrated regulatory potential in lipid and cholesterol metabolism.

Peroxisome proliferator-activated receptors (PPARs) make up a family of nuclear hormone receptors that can be activated by specific ligands. This family comprises three subtypes: PPAR-α, PPAR-γ, and PPAR-β/δ.^9^ Previous studies indicated that activation of PPAR-α and PPAR-γ by specific agonists ameliorates obesity by enhancing energy expenditure.^10^ In fact, several clinically used synthetic ligands targeting PPARα and PPARγ have been developed, while ligands for PPARδ are still under investigation and development.^11^ Several unsaturated fatty acids, fatty acid derivatives, and their mimetics have been reported to act as partial agonists of PPARs, thereby contributing to the alleviation of metabolic dysfunction.^12,13^ More importantly, our previous findings suggested that octacosanol supplementation may improve hepatic lipid accumulation, at least in part, through the upregulation of PPARα expression.^8^

Building upon our previous findings, and considering the limited research on the antiobesity effects of esterified octacosanol, particularly in adipose tissue, this research was designed to fill this gap. We aimed to investigate the beneficial effects of NO, OEO, and LEO on adipose tissue in HFD-fed mice and to elucidate the underlying molecular mechanisms involved.

## Materials and Methods

### Chemicals and Drugs

Octacosanol (C-28, purity >90%; comprising 95% octacosanol [C-28] and 5% triacontanol [C-30]) was purchased from Biosynth Ltd. (Compton, United Kingdom). Dodecanoic acid (purity >99%), oleic acid (purity >99%), and octacosanol standard (≥99%) were obtained from Sigma-Aldrich Co. (St. Louis, MO). Lovastatin (purity >97%) was purchased from Thermo Fisher Scientific Inc. (Waltham, MA). The list of antibody suppliers is presented in Figure S1.

### Esterification of Octacosanol Fatty Acid Derivatives

LEO and OEO were synthesized according to the method outlined in our previous study.^8^ Lauric acid-esterified octacosanol (LEO) and oleic acid-esterified octacosanol (OEO) were synthesized by using purified octacosanol (containing 95% octacosanol and 5% triacontanol) and their respective fatty acids. The molar ratios used were 1:3 (octacosanol/lauric acid, w/w) for LEO and 2:1 (octacosanol/oleic acid, w/w) for OEO. Sulfuric acid was added as a catalyst at 0.5% (v/w) of the combined weight of octacosanol and fatty acid. The esterification reaction was carried for LEO and OEO at 140 °C and 150 °C, respectively, with a reaction time of 6 h (Figure 1). For purification, the esterified products were neutralized to pH 7 using potassium hydroxide (KOH) dissolved in an ethanol-distilled water solution at 30 °C for 1 h. The neutralized mixture was then subjected to a liquid–liquid extraction using distilled water and heptane for phase separation. The samples were further analyzed by FTIR to confirm the conversion of octacosanol into its esterified forms.

**Figure 1:**
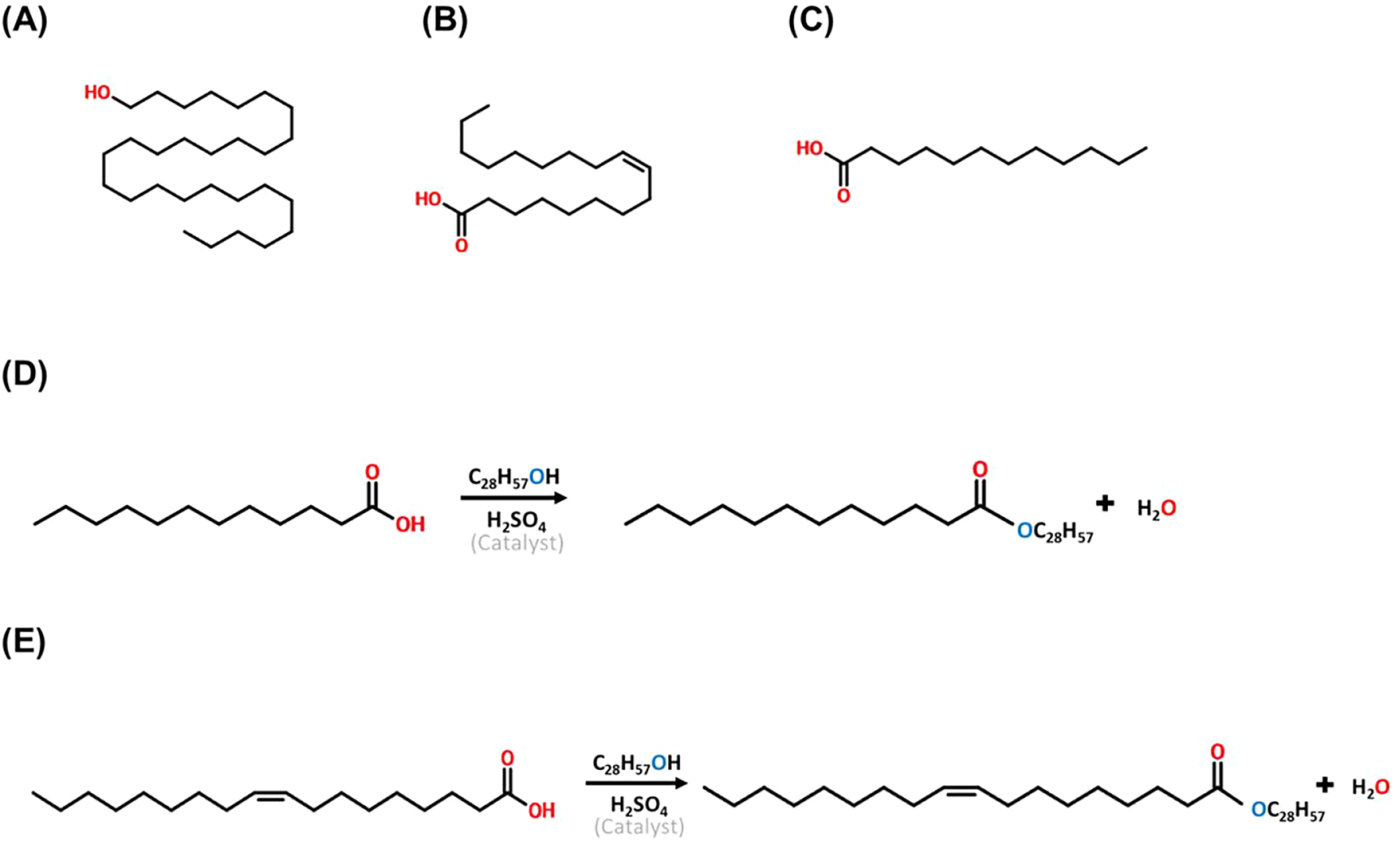
Chemical structures of octacosanol and its esterified derivatives (OEO and LEO), and schematic representation of the esterification reactions. (A) Chemical structure of octacosanol (C_28_H_58_O), (B) oleic acid (C18:1), and (C) lauric acid (C12:0). (D) Schematic representation of the esterification reaction between octacosanol and lauric acid to produce lauric acid-esterified octacosanol (LEO), and (E) esterification with oleic acid to produce oleic acid-esterified octacosanol (OEO).

### Animal Care and Experimental Design

Male C57BL/6J mice (3 weeks old) were purchased from the National Laboratory Animal Center (Taipei, Taiwan). The mice were housed in a temperature-controlled room (23 ± 3 °C) with 50 ± 10% relative humidity and a 12 h light/dark cycle. During a 1-week acclimatization period, the mice were provided with free access to a standard chow diet (Purina 5001, Lab Diet) and water. All animal procedures were approved by the Institutional Animal Care and Use Committee of the National Taiwan University (NTU-112-EL-00095). After acclimatization, mice were randomly assigned to six groups: CON = chow diet (13.4% energy from fat, *N* = 8); HFD = High-fat diet (45% fat and 5% cholesterol, *N* = 8); NO = HFD + 150 mg/kg/day nonesterified octacosanol, *N* = 8; LEO = HFD + 150 mg/kg/day lauric acid-esterified octacosanol, *N* = 8; OEO = HFD + 150 mg/kg/day oleic acid-esterified octacosanol (OEO), *N* = 8; and LV = HFD + 20 mg/kg/day lovastatin (*N* = 8). Octacosanol, LEO, and OEO were incorporated into the HFD chow by homogeneous mixing, and mice had ad libitum access throughout the 11-week intervention. As no vehicle or gavage was used, dose volume and fasted/fed timing were not applicable. The experimental design is shown in Figure S1. Food intake, water intake, and body weight were recorded twice per week. After 11 weeks of dietary intervention, all animals were sacrificed by CO_2_ asphyxiation. Photographs of the physical appearance were taken immediately after the sacrifice. Blood, liver, kidneys, spleen, gonadal, perirenal, inguinal, and interscapular adipose tissues were collected, weighed, photographed, and subjected to further analysis. Mechanistic analyses were conducted in perigonadal visceral adipose tissue, as this depot is the prototypical and most responsive VAT in murine obesity and provides a standardized and reproducible target for metabolic studies.

### Dosage Information

The dosage of NO, OEO, and LEO (150 mg/kg/day) was calculated by converting the recommended human dosage of 20 mg/day for a 70 kg adult, ensuring clinical relevance and comparability to human supplementation.

### Serum Biochemical Parameter Analysis

Blood samples were centrifuged at 5000 *g*  for 10 min at 4 °C, and the resulting sera were stored at −80 °C until analysis. Serum levels of aspartate aminotransferase (AST), alanine aminotransferase (ALT), triglycerides (TG), total cholesterol (T-CHO), high-density lipoprotein cholesterol (HDL-C), and low-density lipoprotein cholesterol (LDL-C) were measured by the National Laboratory Animal Center (NLAC), Taipei, Taiwan.

### Histological Hematoxylin and Eosin (H&E) Staining

Gonadal adipose tissue was fixed in 10% neutral buffered formalin, followed by dehydration and paraffin embedding. Formalin-fixed, paraffin-embedded (FFPE) tissues were sectioned at 3–5 μm thickness, deparaffinized in xylene and rehydrated through a graded ethanol series prior to staining with hematoxylin and eosin (H&E). Adipocyte size in subcutaneous white adipose tissue was quantified using ImageJ software (Rasband, W.S., U.S. National Institutes of Health, Bethesda, MD). Morphological measurements were taken at 200× magnification.

### Molecular Target Prediction

The potential molecular targets of octacosanol were predicted using the SwissTargetPrediction online platform (http://www.swisstargetprediction.ch/). The SMILES structure of octacosanol was used as an input. The predicted results are summarized in Figure S3 and Table S1. Among the top candidates, peroxisome proliferator-activated receptors (PPARs), specifically PPARα and PPARδ, were identified as potential targets. To further investigate the binding interactions between octacosanol and these targets, molecular docking was performed using the 1-Click Docking tool on Mcule (https://mcule.com). The crystal structures of human PPARα (PDB ID: 1K7L) and PPARδ (PDB ID: 1GWX) were retrieved from the Mcule database. Docking simulations were conducted using default parameters, and the resulting binding poses and docking scores were analyzed to evaluate the potential interactions of octacosanol with PPARα and PPARδ.

### Western Blotting Procedure for Protein Expression Analysis

Gonadal adipose tissues were homogenized for 30 seconds in lysis buffer containing 1 mM EGTA, 137 mM NaCl, 5 mM EDTA, 20 mM Tris base, 10% glycerol, and 1% Triton X-100, supplemented with a protease inhibitor cocktail. The lysates were incubated on ice for 1 h with periodic mixing and then centrifuged at 10,000  *g* for 30 min at 4 °C. Supernatants were collected, and protein concentrations were determined using the Bradford assay (Bio-Rad Protein Assay Dye). Equal amounts of protein were mixed with sample buffer, boiled at 100 °C for 10 min, and subjected to 10–13.5% SDS-PAGE. Proteins were transferred to a polyvinylidene difluoride (PVDF) membrane using transfer buffer at 4 °C for 90 min. Membranes were blocked with 5% blocking solution in 20 mM HCl for 1 h at room temperature and then incubated overnight at 4 °C with primary antibodies diluted in the same blocking solution. Following incubation, membranes were washed three times for 10 min each with TPBS (0.2% Tween-20 in 1× PBS) and incubated with horseradish peroxidase (HRP)-conjugated secondary antibodies for 1 h at room temperature. Protein bands were visualized using enhanced chemiluminescence (ECL) and quantified using ImageJ software (Rasband, Washington, DC. U.S. National Institutes of Health, Bethesda, MD).

### Statistical Analysis

Data are presented as mean ±  standard error (SE) from at least triplicate analyses. Differences among group means were assessed using one-way analysis of variance (ANOVA), followed by Tukey’s post hoc test. For comparisons between two groups, the student’s *t* test was used. Statistical analyses were performed using SPSS software (version 22.0; IBM Corp., Armonk, NY). A *p*-value <  0.05 was considered statistically significant.

## Results

### Octacosanol and Esterified Octacosanol Samples Significantly Reduced Weight Gain Induced by HFD Feeding

Changes in physiological and organ parameters are shown in Table 1, and the representative appearances of each group are shown in Figure S2. There were no significant differences in the initial body weight among the groups (Table 1). After 11 weeks, the body weight of mice fed a high-fat diet (HFD) significantly increased to 35.14  ±  1.71 g. Supplementation with nonesterified octacosanol (NO) and oleic acid-esterified octacosanol (OEO) effectively prevented weight gain without reducing food intake. Notably, there were no significant differences in body weight between any of the treatment groups and the positive control group treated with lovastatin. Food efficiency was significantly elevated in the HFD group but was markedly reduced by all supplementations.

**Table 1:** Changes in Physiological and Organ Parameters in Response to Octacosanol Supplementation in HFD-fed Mice^a^,^b^

	CON	HFD	NO	LEO	OEO	LV
initial weight (g)	20.16 ± 0.94 ^a^	20.55 ± 0.83 ^a^	19.96 ± 1.02 ^a^	19.05 ± 1.91 ^a^	19.78 ± 0.79 ^a^	19.90 ± 0.95 ^a^
final weight (g)	29.03 ± 1.67 ^c^	35.14 ± 1.71 ^a^	31.34 ± 1.84 ^b^	31.90 ± 0.97 ^b^	31.25 ± 1.21 ^bc^	31.70 ± 1.46 ^b^
weight gained (g)	8.86 ± 2.11 ^c^	14.59 ± 2.03 ^a^	10.34 ± 3.32 ^bc^	11.79 ± 3.65 ^ab^	10.61 ± 2.85 ^bc^	10.97 ± 3.03 ^b^
food intake (g/day)	5.15 ± 1.01 ^ab^	4.67 ± 0.94 ^b^	5.12 ± 0.57 ^b^	5.12 ± 0.89 ^ab^	6.15 ± 0.58 ^a^	5.07 ± 0.50 ^b^
water intake (g/day)	5.03 ± 0.44 ^a^	4.51 ± 0.25 ^a^	4.95 ± 0.71 ^a^	5.02 ± 0.77 ^a^	4.74 ± 0.29 ^a^	4.56 ± 0.47 ^a^
food efficiency (%)	1.72 ± 0.41 ^d^	3.13 ± 0.43 ^a^	2.02 ± 0.64 ^bcd^	2.50 ± 0.37 ^b^	1.70 ± 0.53 ^cd^	2.13 ± 0.69 ^bc^
liver weight (g)	1.42 ± 0.11 ^b^	1.66 ± 0.07 ^a^	1.26 ± 0.09 ^bc^	1.22 ± 0.03 ^c^	1.14 ± 0.16 ^c^	1.22 ± 0.12 ^c^
kidney weight (g)	0.38 ± 0.03 ^b^	0.45 ± 0.07 ^a^	0.38 ± 0.01 ^b^	0.38 ± 0.02 ^b^	0.35 ± 0.03 ^b^	0.36 ± 0.03 ^b^
spleen weight (g)	0.07 ± 0.01 ^a^	0.08 ± 0.01 ^a^	0.06 ± 0.01 ^a^	0.07 ± 0.01 ^a^	0.07 ± 0.02 ^a^	0.07 ± 0.01 ^a^

a Data are presented as mean ± SD (*N* = 8 independent biological replicates per group).

b Different lowercase letters indicate significant differences among groups, as determined by ANOVA followed by Tukey’s post hoc test.

In terms of organ weight, both liver and kidney weights were significantly increased in the HFD group compared to those in the normal diet (CON) group (Figure 2S). However, this effect was effectively reversed by all supplementations, as shown in Table 1.

**Figure 2:**
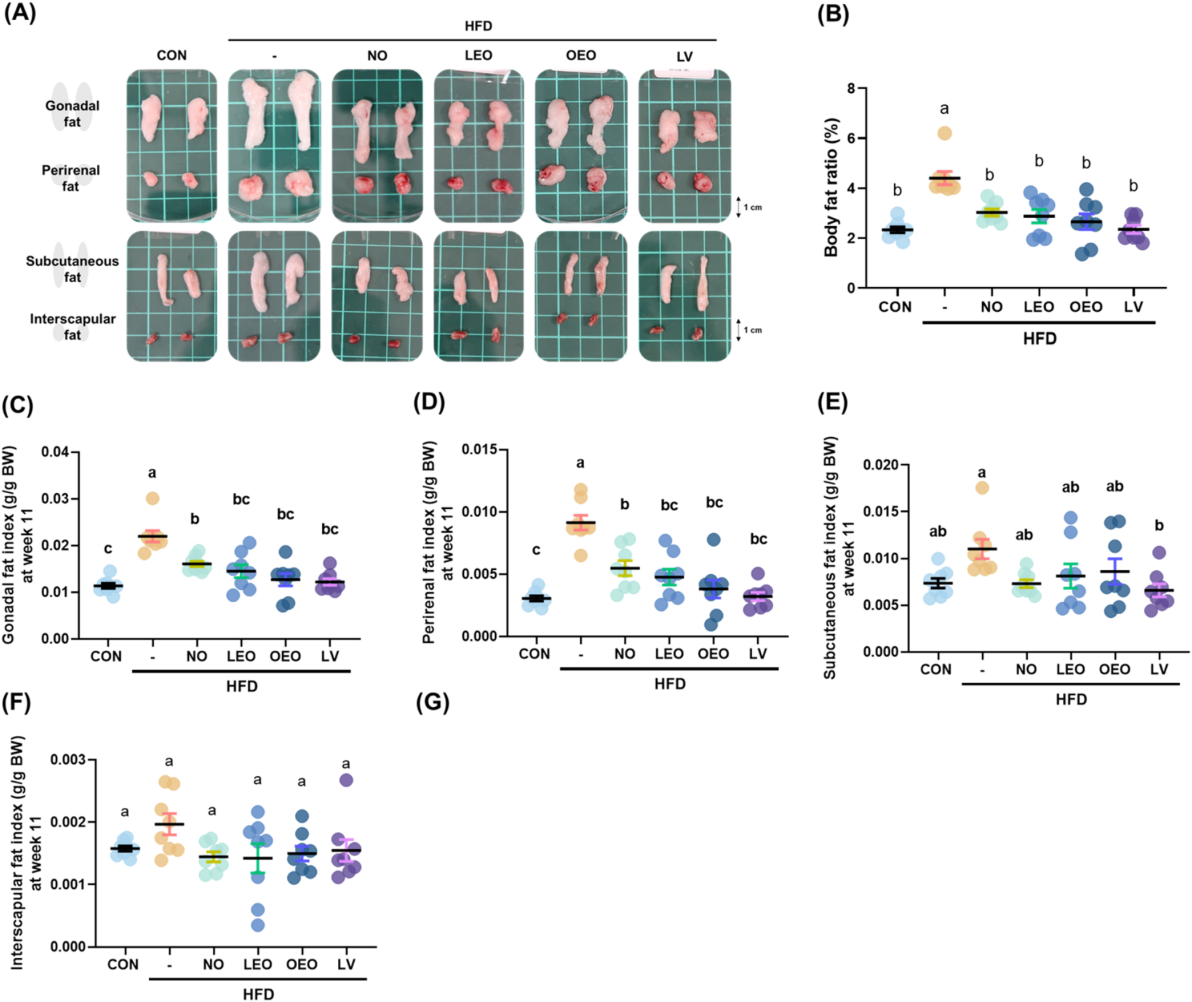
Octacosanol and its esterified forms prevented lipid accumulation in the adipose tissues of HFD-fed mice. (A) Representative images of gonadal, perirenal, subcutaneous, and interscapular adipose tissues from each group. (B) Body fat ratio (%) calculated as the sum of adipose tissue weights divided by final body weight × 100. (C–F) Average weights of (C) gonadal fat, (D) perirenal fat, (E) subcutaneous fat, and (F) interscapular fat. *N* = 8 independent biological replicates per group. Different lowercase letters indicate significant differences among groups, as determined by one-way ANOVA followed by Tukey’s post hoc test (*p* < 0.05).

### Effects of Octacosanol and Esterified Octacosanols on Biochemical Parameters in Different Groups

Serum AST levels and the AST/ALT ratio were significantly increased in the HFD group (Table 2). However, only AST levels were effectively reduced by octacosanol, LEO, OEO, and LV, associated with the observed changes in liver weight. Serum triglyceride (TG) and total cholesterol (T-CHO) levels were also markedly elevated in the HFD group and significantly attenuated by all supplementation treatments.

**Table 2:** Changes in Biochemical Parameters in Response to Octacosanol Supplementation in HFD-Fed Mice^a^,^b^

	CON	HFD	NO	LEO	OEO	LV
AST (U/L)	170.10 ± 33.53^b^	273.27 ± 40.23^a^	220.70 ± 49.50^ab^	189.34 ± 29.21^b^	181.29 ± 12.17^b^	182.17 ± 34.08^b^
ALT (U/L)	33.66 ± 7.47^a^	35.68 ± 6.10^a^	31.17 ± 5.09^a^	32.69 ± 3.18^a^	30.78 ± 4.14^a^	29.00 ± 2.97^a^
AST/ALT	5.16 ± 1.17^b^	7.70 ± 0.51^a^	7.24 ± 2.01^a^	5.86 ± 1.17^ab^	6.00 ± 1.04^ab^	6.34 ± 1.33^ab^
TG (mg/dL)	91.36 ± 5.08^e^	170.70 ± 8.81^a^	141.60 ± 7.56^b^	124.53 ± 6.70^c^	115.00 ± 6.48^cd^	111.80 ± 5.69^d^
T-CHO (mg/dL)	25.43 ± 5.97^b^	71.14 ± 11.68^a^	24.84 ± 7.84^b^	23.16 ± 6.37^b^	16.00 ± 4.28^b^	14.90 ± 5.47^b^
HDL-c (mg/dL)	77.16 ± 3.05^c^	87.33 ± 8.24^cd^	103.99 ± 5.77^bc^	111.36 ± 5.51^ab^	117.74 ± 4.40^a^	100.07 ± 4.42^c^
LDL-c (mg/dL)	9.97 ± 0.69^d^	69.84 ± 2.88^a^	36.69 ± 7.15^b^	31.47 ± 5.24^bc^	26.09 ± 4.36^c^	35.79 ± 3.58^b^
HDL-c/LDL-c	7.77 ± 0.64^a^	1.25 ± 0.15^d^	2.95 ± 0.73^c^	3.67 ± 0.90^bc^	4.61 ± 0.65^b^	2.83 ± 0.39^c^

a Data are presented as mean ± SD (N = 8 independent biological replicates per group).

b Different lowercase letters indicate significant differences among groups, as determined by ANOVA followed by Tukey’s post hoc test.

Notably, OEO and LV were the most effective in lowering serum T-CHO levels, followed by LEO and NO. All treatments significantly increased serum HDL-C levels, with the greatest effects observed in the LEO and OEO groups, followed by NO and LV. Similarly, the OEO showed the strongest effect in reducing serum LDL-C levels compared to the other treatments. The HFD group exhibited the lowest HDL-C/LDL-C ratio among all groups, which was effectively increased by supplementation with the supplemental aqueous OEO, followed by LEO, NO, and LV.

### Octacosanol and its Esterified Octacosanol Prevented Lipid Accumulation in the Adipose Tissues of HFD-Fed Mice

Four different adipose tissues, including gonadal, perirenal, subcutaneous, and interscapular fat, were collected for analysis. The representative images of these tissues are shown in Figure 2A. Marked enlargement of adipose tissues was observed in the HFD group, which was partially reversed by the supplementation treatments. The body fat ratio was calculated by summing the weights of the collected adipose tissues and dividing by the final body weight (Figure 2B). This ratio was significantly increased in the HFD group but was reduced by all of the supplementation treatments. A significant increase in the weight of various adipose tissues, except interscapular fat, was observed in the HFD group. All treatments effectively reduced the weights of gonadal and perirenal fat, while only LV significantly decreased subcutaneous fat compared to the HFD group.

The size distribution, average size, and histological staining of gonadal adipocytes are listed in Figure 3. Compared with other groups, the HFD group exhibited a significantly low proportion of adipocytes within the 501–1000 μm^2^ range and a high proportion within the 2501–5000 μm^2^ range. These findings were consistent with the average adipocyte size shown in Figure 3B, and the representative H&E-stained images in Figure 3C, both indicating that the HFD group had significantly larger adipocytes than the other groups.

**Figure 3:**
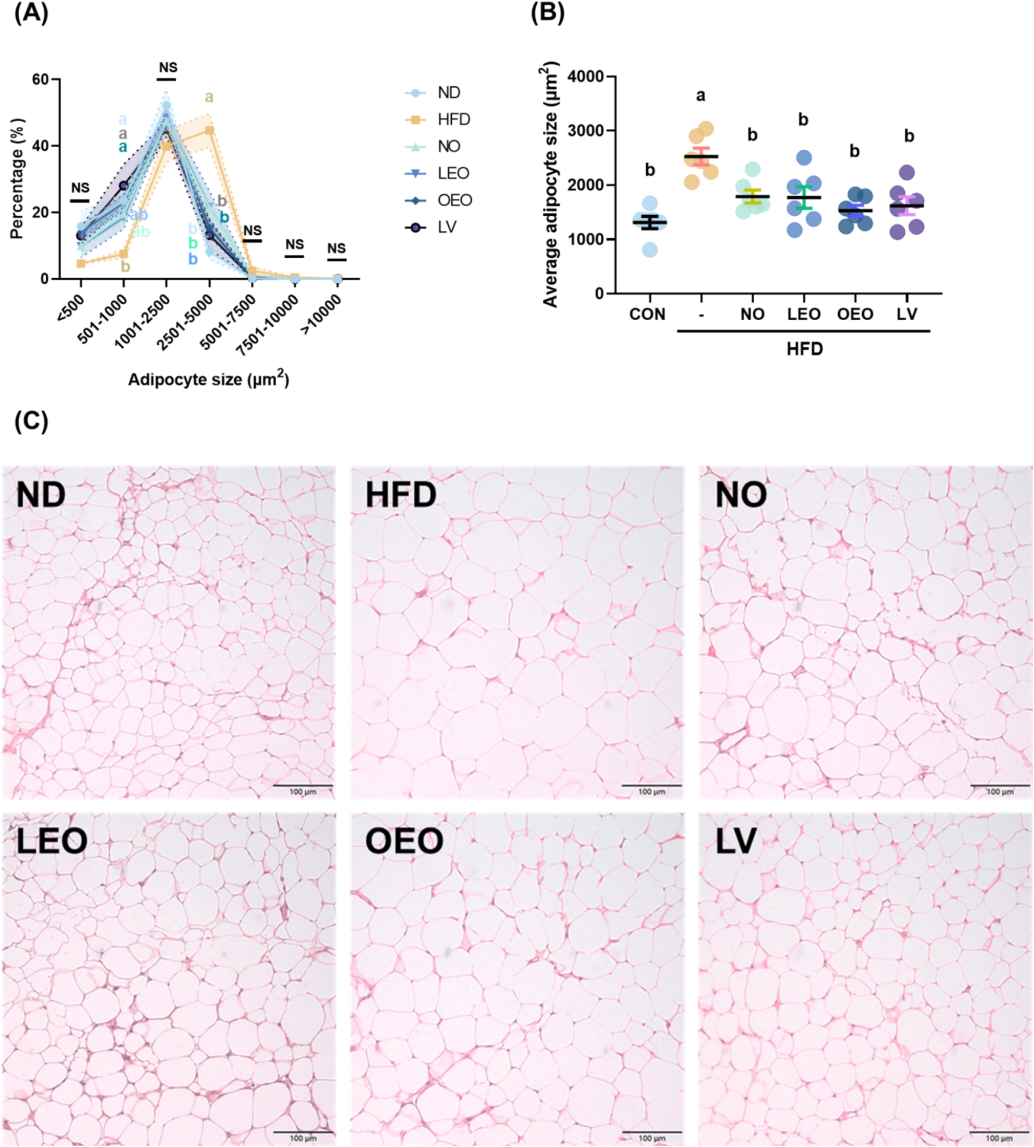
Effects of octacosanol and its derivatives on gonadal adipocyte size and morphology in HFD-fed mice. (A) Adipocyte size distribution in gonadal adipose tissue from each group. (B) Average adipocyte size (μm^2^). (C) Representative H&E-stained images of gonadal adipose tissue sections (200× magnification). *N* = 6 independent biological replicates per group. Different lowercase letters indicate significant differences among groups, as determined by one-way ANOVA followed by Tukey’s post hoc test (*p* < 0.05).

### PPAR-α and PPAR-δ Were the Potential Molecular Targets of Octacosanol

The potential molecular targets of octacosanol were predicted using SwissTargetPrediction. Among the 38 predicted targets, 40% of the top 15 were classified as nuclear receptors (Figure S3). Notably, among these nuclear receptors, peroxisome proliferator-activated receptor α (PPARα) and peroxisome proliferator-activated receptor delta (PPARδ) are nuclear transcription factors closely associated with lipid metabolism, particularly fatty acid oxidation (Table S2).

To further explore these interactions, molecular docking analysis was performed using Mcule.com with octacosanol as the ligand. The highest docking scores for octacosanol binding to PPARδ and PPARα were −6.8 and −6.4, respectively (Figure 4A,D).

**Figure 4:**
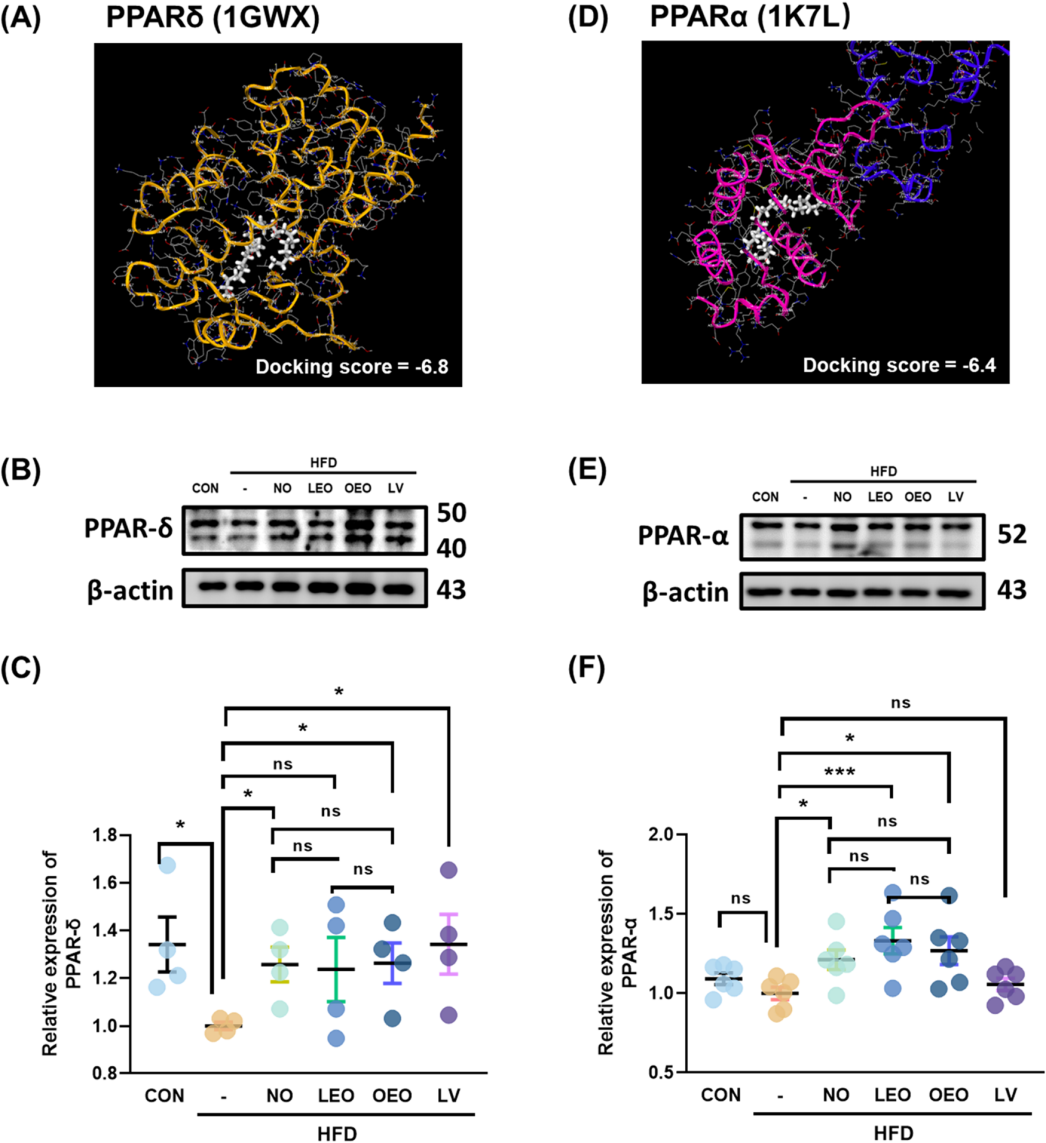
Molecular docking prediction and expression of PPARα and PPARδ in gonadal adipose tissue. (A) Docking pose of octacosanol with human PPARδ (PDB ID: 1GWX), generated using the Mcule 1-Click Docking tool. (B) Representative Western blot image and (C) quantification of PPARδ protein expression in gonadal adipose tissue. (D) Docking pose of octacosanol with human PPARα (PDB ID: 1K7L), generated using the Mcule 1-Click Docking tool. (E) Representative Western blot image and (F) quantification of PPARα protein expression in gonadal adipose tissue. *N* = 4–6 independent biological replicates per group. Asterisks (*) and (**) indicate significant differences compared to the HFD group, with *p* < 0.05 and *p* < 0.01, respectively, as determined by Student’s *t* test.

To validate these predictions, the protein expression levels of PPARδ and PPARα in gonadal adipose tissue were assessed by Western blot analysis. Both nonesterified octacosanol (NO) and oleic acid-esterified octacosanol (OEO) significantly upregulated the expression of PPARδ and PPARα (Figure 4B,C,E,F), whereas lauric acid-esterified octacosanol (LEO) increased PPARα expression but had no effect on PPARδ.

### Octacosanol and Esterified Octacosanol Promoted Lipolytic Enzyme Activity via PPAR-α Activation

HSL and ATGL are downstream enzymes regulated by PPARα. Therefore, the protein expression levels of these lipolytic enzymes were assessed. Our results showed that both NO and OEO significantly increased the expression of HSL and ATGL (Figure 5A,D,E). Notably, LEO upregulated ATGL but had no effect on HSL expression.

**Figure 5:**
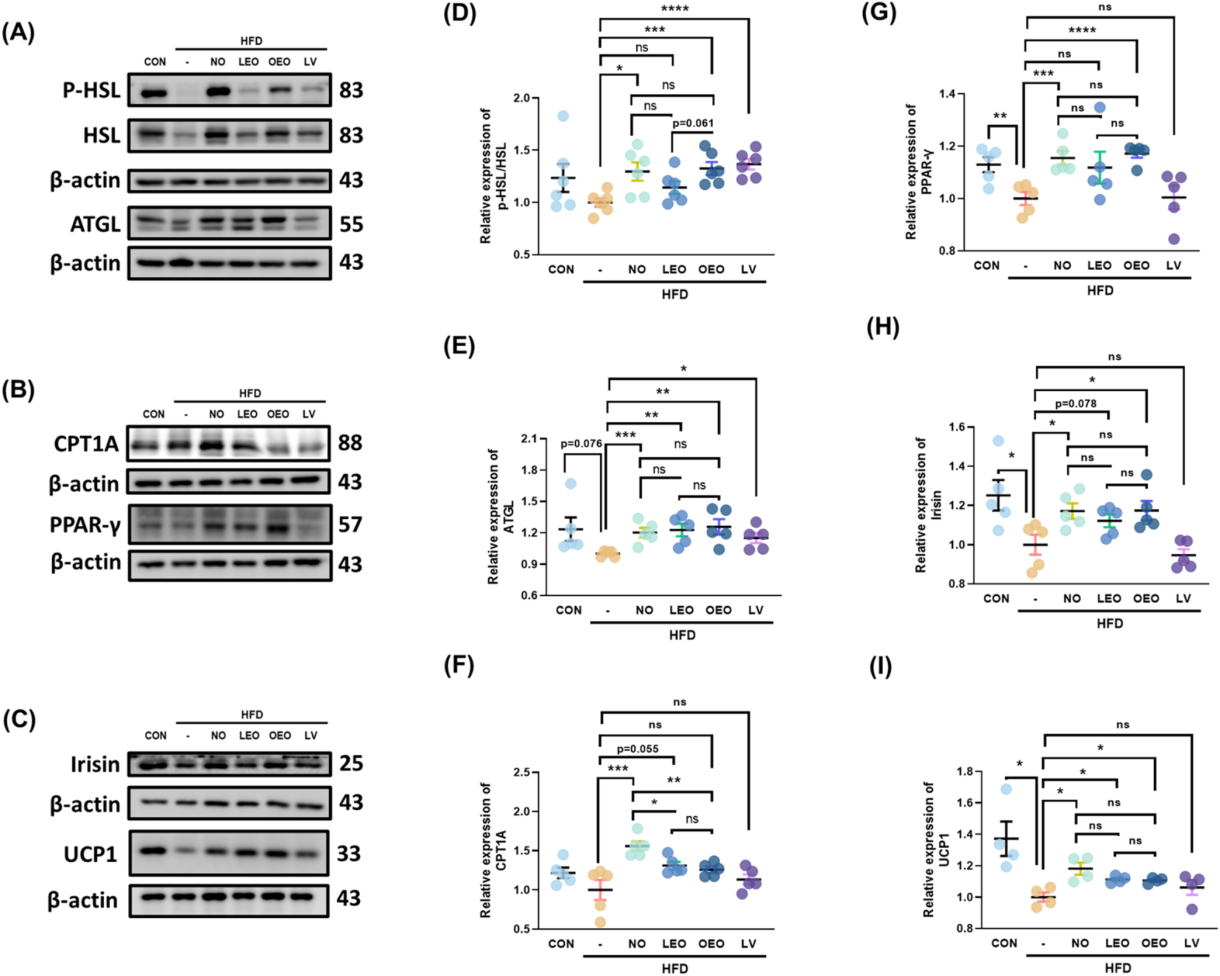
Effects of octacosanol and its derivatives on PPAR-related downstream proteins in gonadal adipose tissue. (A–C) Representative Western blot images of the following proteins in gonadal adipose tissue: (A) phosphorylated HSL (p-HSL)/total HSL and ATGL, (B) CPT1A and PPARγ, and (C) irisin and UCP1. (D–I) Quantification of protein expression levels for: (D) p-HSL/HSL, (E) ATGL, (F) CPT1A, (G) PPARγ, (H) irisin, and (I) UCP1. *N* = 5–6 independent biological replicates per group. Asterisks (*, **, ***, ****) indicate significant differences compared to the HFD group, with *p* < 0.05, 0.01, 0.005, and 0.001, respectively, as determined by Student’s *t* test.

CPT1A, another downstream target of PPARα and PPARδ involved in β-oxidation, was significantly induced by NO supplementation; however, neither LEO nor OEO promoted CPT1A expression (Figure 5B,F). Although PPARγ, another nuclear transcription factor upstream of CPT1A, was not predicted to be a molecular target of octacosanol, both NO and OEO were found to increase its level of protein expression (Figure 5B,G).

PPARs play essential roles in adipose tissue browning and thermogenesis. Irisin, a protein involved in browning, has been reported to be indirectly regulated by both PPARα and PPARδ. Our results demonstrated that NO and OEO enhanced irisin protein levels in gonadal white adipose tissue. Furthermore, UCP1, a key thermogenic protein regulated by PPARs, was upregulated following all supplementations (Figure 5C,H,I). These findings suggest that all treatments, particularly NO and OEO, effectively reduce adipose tissue expansion potentially by promoting both lipolysis and thermogenesis.

### Reducing Lipid Synthesis of Octacosanol and Esterified Octacosanol by Activated AMPK

In addition to PPARs, we investigated the effects of the samples on AMP-activated protein kinase (AMPK), which is another key regulator of lipid metabolism and synthesis. In the HFD group, AMPK activation was significantly suppressed, whereas all treatment groups, including the positive control (lovastatin), effectively restored AMPK activity. Expression of SIRT1 was promoted by all samples but only OEO could increase the protein level of PGC-1α (Figure 6A,E,F). Activation of AMPK led to increased phosphorylation of acetyl-CoA carboxylase (ACC), thereby inhibiting fatty acid synthesis (Figure 6A,G).

**Figure 6:**
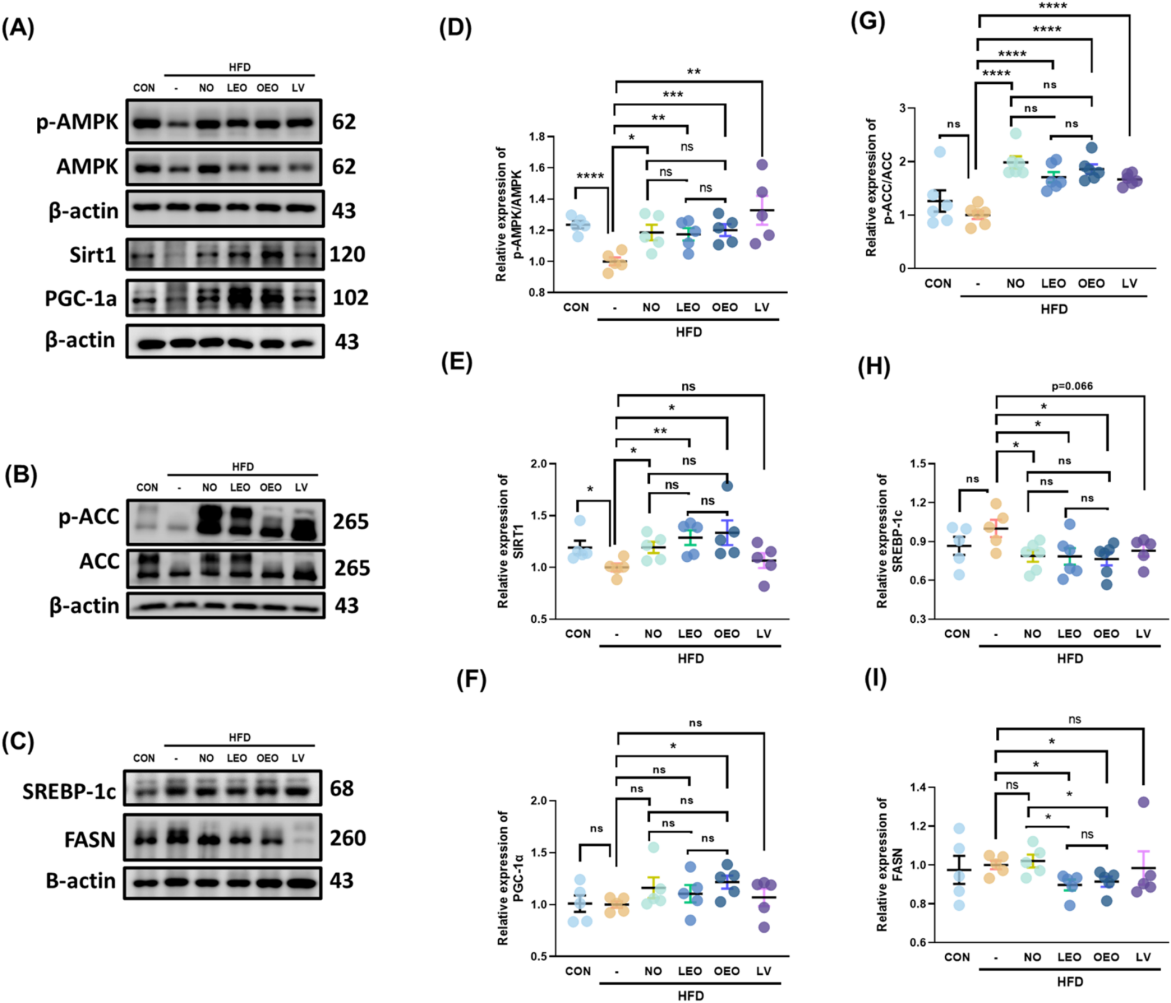
Effects of octacosanol and its derivatives on AMPK-mediated pathway in gonadal adipose tissue. (A–C) Representative Western blot images of the following proteins in gonadal adipose tissue: (A) phosphorylated AMPK (p-AMPK)/total AMPK, SIRT1, and PGC-1α, (B) phosphorylated ACC (p-ACC)/total ACC, and (C) SREBP-1c and FASN. (D–G) Quantification of protein expression levels for: (D) p-AMPK/AMPK, (E) SIRT1, (F) PGC-1α, (G) p-ACC/ACC, (H) SREBP-1c, and (I) FASN. *N* = 5–8 independent biological replicates per group. Asterisks (*, **, ***, ****) indicate significant differences compared to the HFD group, with *p* < 0.05, 0.01, 0.005, and 0.001, respectively, as determined by Student’s *t* test.

Consistent with this, the levels of cleaved SREBP-1c, a transcription factor involved in lipogenesis, were significantly reduced in all sample groups compared to those in the HFD group. Furthermore, expression of fatty acid synthase (FASN) was markedly decreased in the LEO and OEO groups, supporting the antilipogenic effects of these treatments (Figure 6).

### The Effect of Octacosanol and Esterified Octacosanol on Cholesterol Biosynthesis Pathway in Adipose Tissue

Although adipose tissue is not the primary site of cholesterol biosynthesis, it possesses the enzymatic machinery necessary for cholesterol production, particularly under conditions of metabolic stress. In light of lovastatin’s well-established inhibitory effect on this pathway, we further investigated whether the tested treatments could modulate the expression of cholesterol biosynthesis-related enzymes.

Our results showed that all treatments, including lovastatin, significantly reduced the cleaved form of SREBP2, a master regulator of cholesterol biosynthesis (Figure 7). As a key transcription factor, SREBP2 regulates several downstream targets, including LDLR, HMGCS, and HMGCR, the primary targets of lovastatin, all of which were significantly down-regulated in expression. Notably, among the tested treatments, only LEO reduced the expression of LSS and FDFT1. These findings suggest that the potential inhibitory effect of LEO on cholesterol biosynthesis warrants further investigation in future studies.

**Figure 7:**
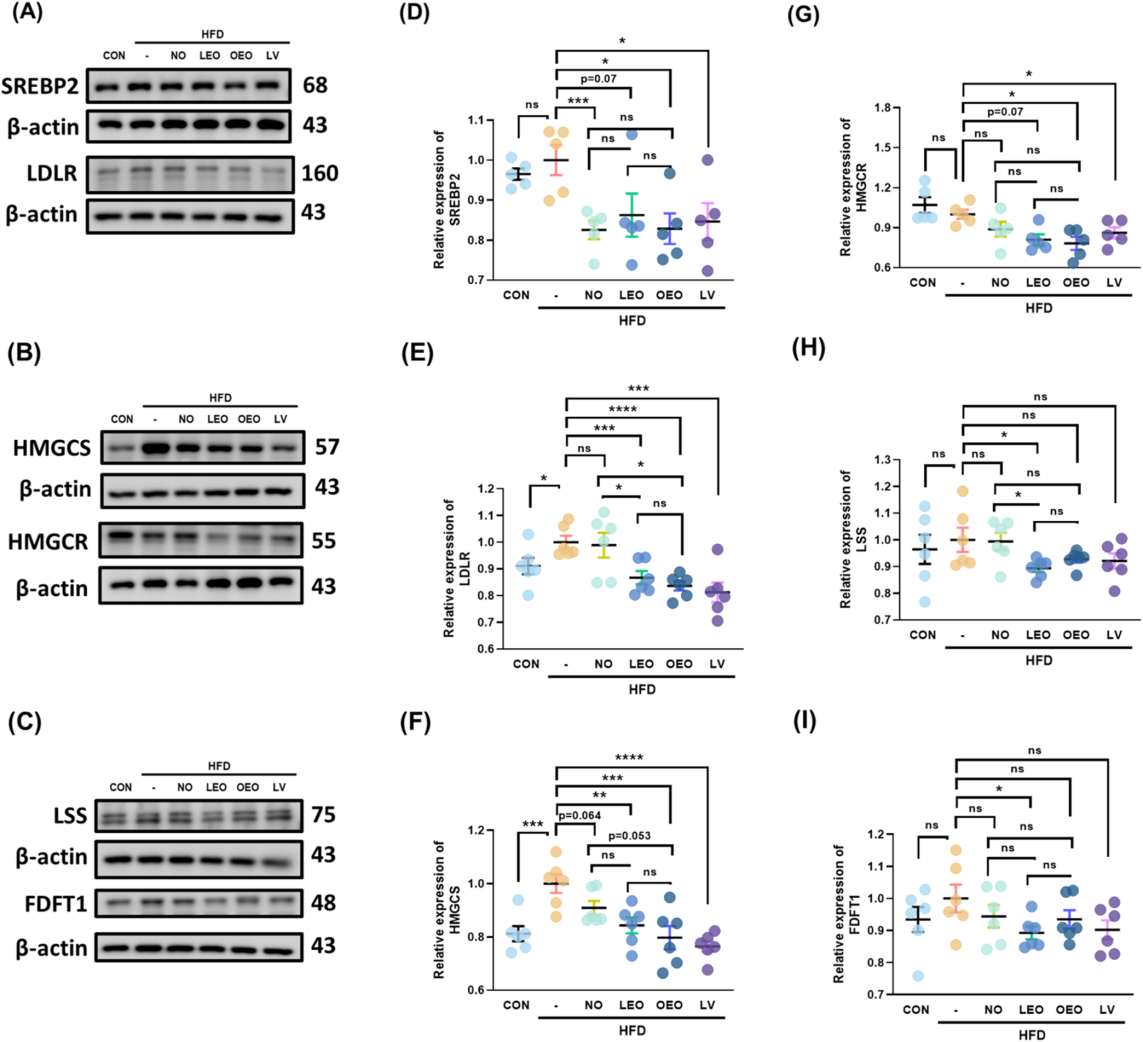
Effects of octacosanol and its derivatives on cholesterol biosynthesis-related pathway in gonadal adipose tissue. (A–C) Representative Western blot images of the following proteins in gonadal adipose tissue: (A) SREBP-2 and LDLR, (B) HMGCS and HMGCR, and (C) LSS and FDFT. (D–I) Quantification of protein expression levels for: (D) SREBP2, (E) LDLR, (F) HMGCS, (G) HMGCR, (H) LSS, and (I) FDFT1. *N* = 5–6 independent biological replicates per group. Asterisks (*, **, ***, ****) indicate significant differences compared to the HFD group, with *p* < 0.05, 0.01, 0.005, and 0.001, respectively, as determined by Student’s *t* test.

## Discussion

This study aimed to evaluate the antiobesity property of octacosanol and fatty acid-esterified octacosanol and to elucidate their underlying molecular mechanisms. Our findings demonstrated that supplementation with NO, LEO, and OEO significantly reduced body weight and adipose tissue expansion induced by high-fat diet (HFD) feeding. Molecular target prediction using SwissTargetPrediction identified PPARα and PPARδ as potential targets of octacosanol. Subsequent Western blot analysis confirmed that several downstream targets of PPARα and PPARδ, particularly those involved in β-oxidation and thermogenesis, were significantly upregulated. Additionally, supplementation, especially with NO and OEO, enhanced the AMPK pathway activation and reduced the expression of key proteins involved in cholesterol biosynthesis.

In 2019, it was demonstrated that octacosanol treatment in chow-fed mice significantly increased the mRNA levels of thermogenesis- and energy expenditure-related genes, including *Ucp1* and *Ppara*, in both brown adipose tissue and inguinal white adipose tissue.^14^ Our findings are consistent with these observations. Although gonadal adipose tissue is traditionally considered to be resistant to browning, supplementation with OEO promoted the expression of UCP1 (Figure 5). This effect was linked to the increased protein expression of PPARα and PPARγ induced by the supplementation. Previous studies have reported that activation of PPARα or dual PPARα/γ agonists can stimulate UCP1-dependent pathways in beige adipocytes, thereby supporting mitochondrial metabolism and maintaining mitophagy capacity.^15^

Our findings also indicated that the expression levels of HSL and ATGL were upregulated, suggesting a lipolytic response induced by the treatments (Figure 5A). Importantly, upregulation of ATGL has previously been observed following PC supplementation in the inguinal adipose tissue of HFD-fed mice.^16^ While ATGL activity is commonly associated with PPARγ activation, growing evidence suggests that PPARα and PPARδ also exert tissue-specific regulatory effects on ATGL-mediated lipolysis.^17^ Regarding β-oxidation, CPT1A expression was significantly increased in the NO group (Figure 5B). As a key enzyme in fatty acid β-oxidation, CPT1A is directly regulated by PPARs.^18^ In the PNAS study, activation of PPAR-δ was shown to enhance mitochondrial biogenesis and fatty acid oxidation programs in adipose tissues, supporting the notion that upregulation of PPAR-δ downstream targets (including CPT1A and UCP1) is mechanistically plausible in our system.^19^

In contrast, LEO and OEO supplementation showed only nonsignificant upward trends. Despite similar PPAR upregulation by NO and OEO in gonadal adipose tissue, downstream responses demonstrated pathway bias rather than a contradiction. NO preferentially activated the PPARα-CPT1A axis, whereas OEO primarily enhanced thermogenic/browning markers, including UCP1 and irisin, while NO also elevated irisin (Figure 5). We therefore propose that the esterified form (OEO) biases signaling toward thermogenesis/browning via combined PPARα/δ (and increased PPARγ) activity in this depot, whereas the nonesterified form (NO) more strongly engages the PPARα-β-oxidation pathway (CPT1A). Previous studies have shown that PPAR-β/δ activation preferentially promotes fatty acid oxidation and metabolic remodeling in white adipose depots, although its capacity to drive full thermogenic browning via UCP1 induction may depend on depot type and experimental context.^20^ This interpretation is consistent with the influence of coregulators (e.g., PGC-1α) and depot-specific signaling on determining which PPAR-dependent programs predominate (Figure 6). The modest but nonsignificant increase in CPT1A observed with OEO may also reflect differences in time-course or transcriptional sensitivity compared with protein end points in gonadal fat. Collectively, these findings indicate mechanistic divergence in downstream pathway preference rather than inconsistency, and we highlight this as a limitation warranting future time-resolved and depot-comparative analyses.

In our previous study, hepatic AMPK activation was observed in all treatment groups, with the most pronounced effect in the OEO group.^8^ In the present study, all supplementation groups effectively activated AMPK in gonadal adipose tissue (Figure 6A), accompanied by increased phosphorylation of its downstream target ACC (p-ACC/ACC; Figure 6B) and suppression of the lipogenic markers SREBP1c and FASN (Figure 6C). These results support the observed reductions in adipose tissue expansion and adipocyte hypertrophy. Supporting this mechanism, Banerjee et al. (2011) demonstrated that PC enhanced AMPK phosphorylation by more than 2-fold.^21^ Additionally, other studies have suggested that PC inhibits cholesterol biosynthesis via AMPK activation and subsequent deactivation of HMGCR.^22^ It is worth noting that LEO and OEO structurally contain long-chain fatty acids. AMPK activation has been reported in studies involving long-chain fatty acid derivatives, suggesting that esterified compounds serve as potential AMPK activators.^23^ However, further investigation is required to confirm this mechanism and establish a definitive conclusion.

Although adipose tissue is not the primary site of cholesterol synthesis, its synthesis rate has been reported to be approximately 4% of that in the liver.^24^ As noted above, PC has been widely reported to suppress cholesterol biosynthesis, potentially through inhibition of HMGCR.^25^ However, other studies have indicated that PC does not directly inhibit HMGCR and was unable to reduce its expression by more than 50%, even at the highest tested concentrations.^26^ In our study, the protein levels of SREBP2 were significantly reduced in most supplementation groups compared to those in the HFD group (Figure 7). Previous research has shown that AMPK activation can directly inhibit SREBP2 and its downstream targets, including HMGCR and HMGCS.^27^ In addition, irisin has been reported to suppress hepatic cholesterol levels by activating AMPK and inhibiting SREBP2.^28^ Irisin, although primarily secreted by skeletal muscle as a myokine, is also expressed in the adipose tissue. Previous studies have shown that both white and brown fat depots secrete irisin.^29^ We observed that irisin expression was significantly reduced in HFD-fed mice but was markedly elevated following supplementation with NO and OEO (Figure 5). This increase in irisin has partially contributed to AMPK activation and the subsequent downregulation of SREBP2 observed in our results. However, the molecular mechanisms underlying the promotion of irisin expression by these supplements are required to be clarified. Irisin is an adipo-myokine hormone primarily produced by skeletal muscle in response to exercise.^30^ Therefore, the potential effects of octacosanol and its esterified derivatives on skeletal muscle function and irisin production warrant further investigation.

Interestingly, only LEO reduced FDFT1 and LSS, whereas the results of OEO did not. Although we did not find studies directly comparing linoleic- versus oleic-esterified octacosanol on these SREBP2 targets, prior work shows that PUFAs (e.g., linoleic acid) generally exert stronger suppression of SREBP signaling, most prominently SREBP-1, than MUFAs (e.g., oleic acid).^31–33^ These differences provide a plausible, testable explanation that the linoleate moiety in LEO may bias signaling toward stronger repression of the cholesterogenic program, yielding selective FDFT1/LSS downregulation. We emphasize that this remains a mechanistic hypothesis, and future studies will need to quantify nuclear SREBP2, SCAP/INSIG dynamics, and promoter-level regulation of FDFT1/LSS under LEO versus an OEO.

In our study, supplementation with octacosanol and its esterified forms effectively reduced serum triglycerides (TG), total cholesterol (TC), and low-density lipoprotein cholesterol (LDL-C), while increasing high-density lipoprotein cholesterol (HDL-C) levels (Table 2). These findings are consistent with previous studies by Zhai et al. (2021) and Li et al. (2023), which demonstrated that PC supplementation improved serum lipid profiles in HFD-fed mice.^16,34^ In a human study, the percentage of HDL-C was increased in both young smokers and nonsmokers following 8 weeks of PC supplementation, accompanied by improvements in both HDL particle number and size.^35^ Similarly, Cho et al. (2018) reported that PC improved blood pressure, lipid profiles, and HDL functionality in a placebo-controlled trial.^36^ In addition, previous reviews have indicated that Policosanol can reduce serum LDL-C levels by 21–29% in placebo-controlled studies.^37^ The reduced expression of LDLR in gonadal adipose tissue observed in our study represents a compensatory response to the decreased circulating LDL-C levels.

In summary, this study demonstrates that octacosanol and its esterified forms exert beneficial effects on adipose tissue metabolism in HFD-fed mice. The samples showed the most pronounced inhibitory effects on lipid and cholesterol synthesis, likely mediated through AMPK activation and subsequent downregulation of the SREBP-1c, FASN, and SREBP-2 pathways. In addition, both the OEO and NO significantly upregulated the expression of key lipolytic enzymes, including ATGL and HSL, indicating enhanced lipid mobilization. Furthermore, the OEO enhanced adipose tissue thermogenesis by upregulating UCP1 and irisin, suggesting a dual role in promoting lipolysis and energy expenditure. While NO and LEO also exhibited certain regulatory effects on adipose end points, their overall impact was moderate and LEO did not extend to significant changes in body weight gain. These findings highlight the potential of octacosannol and its esterified counterpart as promising functional compounds for managing obesity-related lipid and cholesterol metabolism disorders. Further investigation into the bioavailability, molecular targets, and long-term effects of esterified octacosanol in clinical settings is warranted to support its application in functional foods and therapeutic interventions.

## Limitation

(1) Although equimolar comparisons could provide mechanistic insights, we deliberately adopted a mass-based dosage derived from human clinical supplementation (20 mg/day for a 70 kg adult) to maximize translational relevance. Future studies using equimolar dosing may further complement these findings.
(2) A limitation of this study is that the observed suppression of FDFT1 and LSS was unique to LEO, and while we propose that this may reflect stronger SREBP repression by the linoleate moiety compared with oleate, current evidence directly linking fatty-acid type to these specific SREBP2 targets is scarce. Our interpretation therefore remains a mechanistic hypothesis. Future work is needed to confirm whether linoleic acid-containing derivatives indeed exert stronger repression of cholesterogenic genes than oleic acid derivatives.
(3) Another limitation of this study is that the claims regarding potential protein targets rely in part on molecular docking. While docking provided useful hypothesis-generating insights, it cannot by itself establish direct binding or pathway causality. Future validation using biophysical or biochemical assays (e.g., surface plasmon resonance, isothermal titration calorimetry, coimmunoprecipitation, or reporter assays) will be necessary to confirm target engagement.

## Supplementary Material



## Notes

Predicted molecular targets of octacosanol generated by SwissTargetPrediction (Table S1); list of antibodies suppliers (Table S2); experimental design of the animal study (Figure S1); representative images of mouse appearance and major organs (Figure S2); classification of predicted molecular targets of octacosanol using SwissTargetPrediction (Figure S3) (PDF)
