# Supplementary material for: Oleic Acid-Esterified Octacosanol as a Functional Ingredient to Counter Obesity-Associated Lipid Dysregulation through PPAR-Targeted Regulation
Source: J Agric Food Chem. 2025 Nov 18;74(3):2761–72. doi: 10.1021/acs.jafc.5c10351 (PMC12862754; doi:10.1021/acs.jafc.5c10351)
Supplement: Supplementary file 1 [file jf5c10351_si_001.pdf]

Supporting material

Oleic acid-esterified octacosanol as a functional ingredient to counter obesity-associated lipid dysregulation through PPAR-targeted regulation

Yen-Chun Koh[†,#], Sudthida Kamchonemenukool[‡,#], Pin-Yu Ho[†], Monthana Weerawatanakorn[‡,*], Min-Hsiung Pan[†,§,¶,*]

[†] Institute of Food Science and Technology, National Taiwan University, Taipei 10617, Taiwan

[‡] Department of Agro-Industry, Faculty of Agriculture, Natural Resources and Environment, Naresuan University, 99 Moo 9, Tha Pho, Phitsanulok 65000, Thailand

[§] Department of Medical Research, China Medical University Hospital, China Medical University, 40447 Taichung City, Taiwan

[¶] Department of Health and Nutrition Biotechnology, Asia University, 41354 Taichung City, Taiwan

#These authors contributed equally

* Please send all correspondence to:

Min-Hsiung Pan,

Distinguished Professor

Institute of Food Science and Technology,

National Taiwan University.

No. 1, Sec. 4, Roosevelt Rd., Taipei, 10617, Taiwan

Tel: +886 2 33664133;

E-mail: mhpan@ntu.edu.tw

OR

Monthana Weerawatanakorn,

Associate Professor

Department of Agro-Industry

Faculty of Agriculture Natural Resources and Environment

Naresuan University,

99 Moo 9, Tha Pho, Mueang, Phitsanulok 65000, Thailand

Tel: 66-55-962735

Table S1. Predicted molecular targets of octacosanol generated by SwissTargetPrediction.

Target	Common name	Uniprot ID	ChEMBL ID	Target Class	Known actives (3D/2D)
Transient receptor potential cation channel subfamily M member 8 (by homology)	Trpm8	Q8R4D5	CHEMBL3108632	Voltage-gated ion channel	0 / 1
Androgen Receptor (by homology)	Ar	P19091	CHEMBL3056	Nuclear receptor	0 / 9
Constitutive androstane receptor (by homology)	Nr1i3	O35627	CHEMBL3069	Nuclear receptor	0 / 2
Estrogen receptor alpha (by homology)	Esr1	P19785	CHEMBL3065	Nuclear receptor	0 / 1
Estrogen receptor beta (by homology)	Esr2	O08537	CHEMBL2995	Nuclear receptor	0 / 1
Dual specificity phosphatase Cdc25B (by homology)	Cdc25b	P30306	CHEMBL2723	Phosphatase	0 / 5
G-protein coupled bile acid receptor 1 (by homology)	Gpbar1	Q80SS6	CHEMBL1255150	Family A G protein-coupled receptor	0 / 16
Sonic hedgehog protein	Shh	Q62226	CHEMBL5387	Unclassified protein	0 / 10
11-beta-hydroxysteroid dehydrogenase 1	Hsd11b1	P50172	CHEMBL3910	Enzyme	0 / 5
Niemann-Pick C1-like protein 1 (by homology)	Npc1l1	Q6T3U4	CHEMBL1075296	Other membrane protein	0 / 5
Dipeptidyl peptidase IV (by homology)	Dpp4	P28843	CHEMBL3883	Protease	0 / 1
P-selectin (by homology)	Selp	Q01102	CHEMBL2455	Adhesion	2 / 0
Solute carrier family 22 member 20	Slc22a20	Q80UJ1	CHEMBL5269	Electrochemical transporter	0 / 5
**Peroxisome proliferator-activated receptor alpha (by homology)**	**Ppara**	**P23204**	**CHEMBL2128**	**Nuclear receptor**	**0 / 3**
**Peroxisome proliferator-activated receptor delta (by homology)**	**Ppard**	**P35396**	**CHEMBL2458**	**Nuclear receptor**	**0 / 2**
Sphingosine kinase 1 (by homology)	Sphk1	Q8CI15	CHEMBL2401605	Enzyme	0 / 2
**Free fatty acid receptor 1 (by homology)**	Ffar1	Q76JU9	CHEMBL5411	Family A G protein-coupled receptor	0 / 1
Epoxide hydratase	Ephx2	P34914	CHEMBL4140	Protease	4 / 0
Testosterone 17-beta-dehydrogenase 3 (by homology)	Hsd17b3	P70385	CHEMBL1932905	Enzyme	0 / 11
Cannabinoid CB1 receptor (by homology)	Cnr1	P47746	CHEMBL3037	Family A G protein-coupled receptor	1 / 0
Cannabinoid CB2 receptor (by homology)	Cnr2	P47936	CHEMBL5373	Family A G protein-coupled receptor	1 / 0
Solute carrier family 22 member 6	Slc22a6	Q8VC69	CHEMBL5653	Electrochemical transporter	0 / 1
Ectonucleotide pyrophosphatase/phosphodiesterase family member 2 (by homology)	Enpp2	Q9R1E6	CHEMBL3826871	Enzyme	1 / 0
Dopamine D2 receptor (by homology)	Drd2	P61168	CHEMBL3427	Family A G protein-coupled receptor	1 / 0
Matrix metalloproteinase-2 (by homology)	Mmp2	P33434	CHEMBL3095	Protease	1 / 0
Acetylcholinesterase (by homology)	Ache	P21836	CHEMBL3198	Hydrolase	2 / 0
Group X secretory phospholipase A2 (by homology)	Pla2g10	Q9QXX3	CHEMBL4200	Enzyme	1 / 0
Serine/threonine-protein kinase 12 (by homology)	Aurkb	O70126	CHEMBL1075275	Kinase	1 / 0
Serine/threonine-protein kinase Aurora-A (by homology)	Aurka	P97477	CHEMBL2211	Kinase	1 / 0
Butyrylcholinesterase (by homology)	Bche	Q03311	CHEMBL2528	Hydrolase	1 / 0
Prostanoid EP2 receptor (by homology)	Ptger2	Q62053	CHEMBL2488	Family A G protein-coupled receptor	0 / 1
Prostanoid FP receptor (by homology)	Ptgfr	P43117	CHEMBL5000	Family A G protein-coupled receptor	0 / 1
Cyclooxygenase-1 (by homology)	Ptgs1	P22437	CHEMBL2649	Enzyme	0 / 1
11-beta-hydroxysteroid dehydrogenase 2 (by homology)	Hsd11b2	P51661	CHEMBL3490	Enzyme	0 / 1
DNA polymerase beta (by homology)	Polb	Q8K409	CHEMBL4565	Enzyme	1 / 1
Plasminogen (by homology)	Plg	P20918	CHEMBL1075299	Protease	0 / 1

Table S2. List of antibodies suppliers

**Primary Antibodies**	Supplier
**β-actin**	Sigma
**ACC, p-ACC, CPT-1, FASN, FDFT, HMGCR, HMGCS, LDLR, LSS, PPAR-α, PGC1α, SIRT1, SREBP-2, UCP-1**	Proteintech
**ATGL, SREBP-1c**	Santa cruz
**AMPK, p-AMPK, HSL, p-HSL**	Cell signaling
**Irisin, PPAR-γ**	ireal
**PPAR-δ**	Abclonal

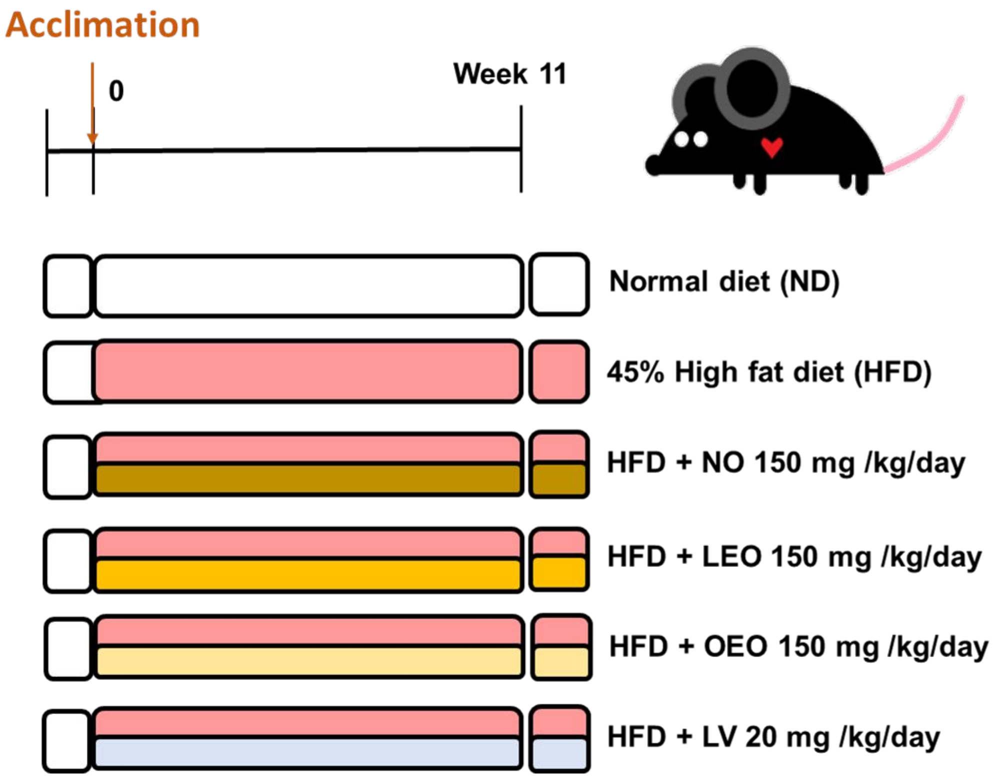

Figure S1. Experimental design of the animal study.

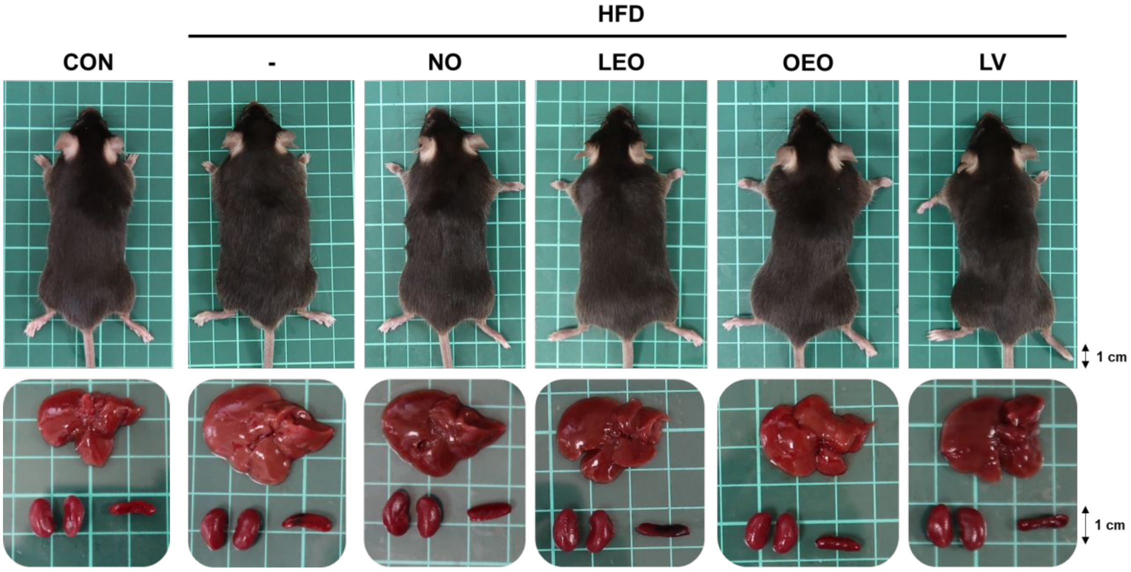

Figure S2. Representative images of mouse appearance and major organs.

Photographs of the overall physical appearance of mice and representative gross morphology of the liver, kidneys, and spleen from each experimental group.

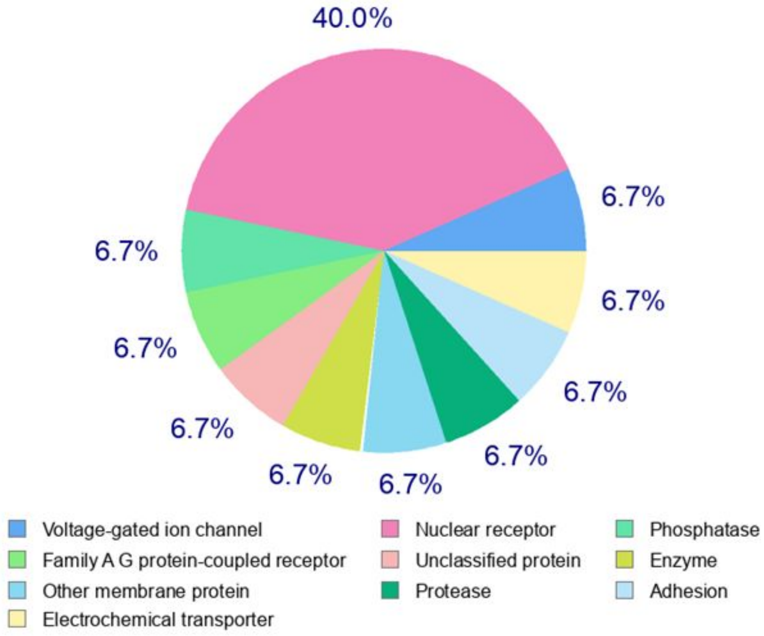

Figure S3. Classification of predicted molecular targets of octacosanol using SwissTargetPrediction

The potential molecular targets of octacosanol were predicted using the SwissTargetPrediction tool. Among the 38 predicted targets, 40% of the top 15 belong to the nuclear receptor class, suggesting possible involvement of transcriptional regulatory pathways.
